# *Codonopsis tangshen* Oliv. Amelioration Effect on Diabetic Kidney Disease Rats Induced by High Fat Diet Feeding Combined with Streptozotocin

**DOI:** 10.1007/s13659-018-0187-5

**Published:** 2018-11-01

**Authors:** Xian-Yuan Lu, Feng-Hua Zhou, Ya-Qian Dong, Lin-Na Gong, Qing-Yun Li, Lan Tang, Zheng Cai, Jing-Yu He, Meng-Hua Liu

**Affiliations:** 1 Guangdong Provincial Key Laboratory of New Drug Screening, School of Pharmaceutical Sciences, Southern Medical University, Guangzhou, 510515 China; 2 School of Traditional Chinese Medicine, Southern Medical University, Guangzhou, 510515 China; 3 Bioengineering Research Centre, Guangzhou Institute of Advanced Technology, Chinese Academy of Sciences, Guangzhou, 511458 China

**Keywords:** Diabetic kidney disease, *Codonopisis tangshen* Oliv., Lipid metabolism, Anti-inflammatory, Anti-fibrotic

## Abstract

**Abstract:**

Diabetic kidney disease (DKD) is the most serious microvascular complication during the development of diabetes with the characterizations of glomerular basement membrane thickening, mesangial expansion, and glomerular sclerosis, eventually leading to end-stage renal disease. This study aimed to investigate the melioration effect of *Codonopisis tangshen* Oliv. (COD) on the DKD model, which was established by unilateral nephrectomy (UN)-high fat diet feeding (HFD) combined with streptozotocin (STZ). After the DKD rats were oral treated with COD at a dose of 2.7 mg/kg for 4 consecutive weeks, the blood glucose, lipid metabolism, renal function, inflammatory mediators, and fibrosis-associated proteins were examined. In vivo, the COD administration obviously relieved the weight loss, water intake, and blood glucose; decreased the total cholesterol, triglyceride, and low-density lipoprotein cholesterol levels; and improved the renal function by reducing the expression of serum creatinine, uric acid, and urinary protein compared with the model group. The levels of pro-inflammatory cytokines of tumor necrosis factor-α, interleukin-1β, and IL-6 were significantly inhibited by COD. Meanwhile, the deposition of collagen fiber was markedly increased, and the protein and mRNA expressions of transforming growth factor-β1 and α-smooth muscle actin were markedly elevated in DKD rats, but they were decreased to some extent after the COD treatment. In conclusion, COD exhibited a protective effect on the UN-HFD feeding combined with STZ-induced DKD model by improving the blood glucose and lipid metabolism, relieving the inflammatory response, and mitigating the renal fibrosis, which provided scientific evidence for its applications in clinic.

**Graphical Abstract:**

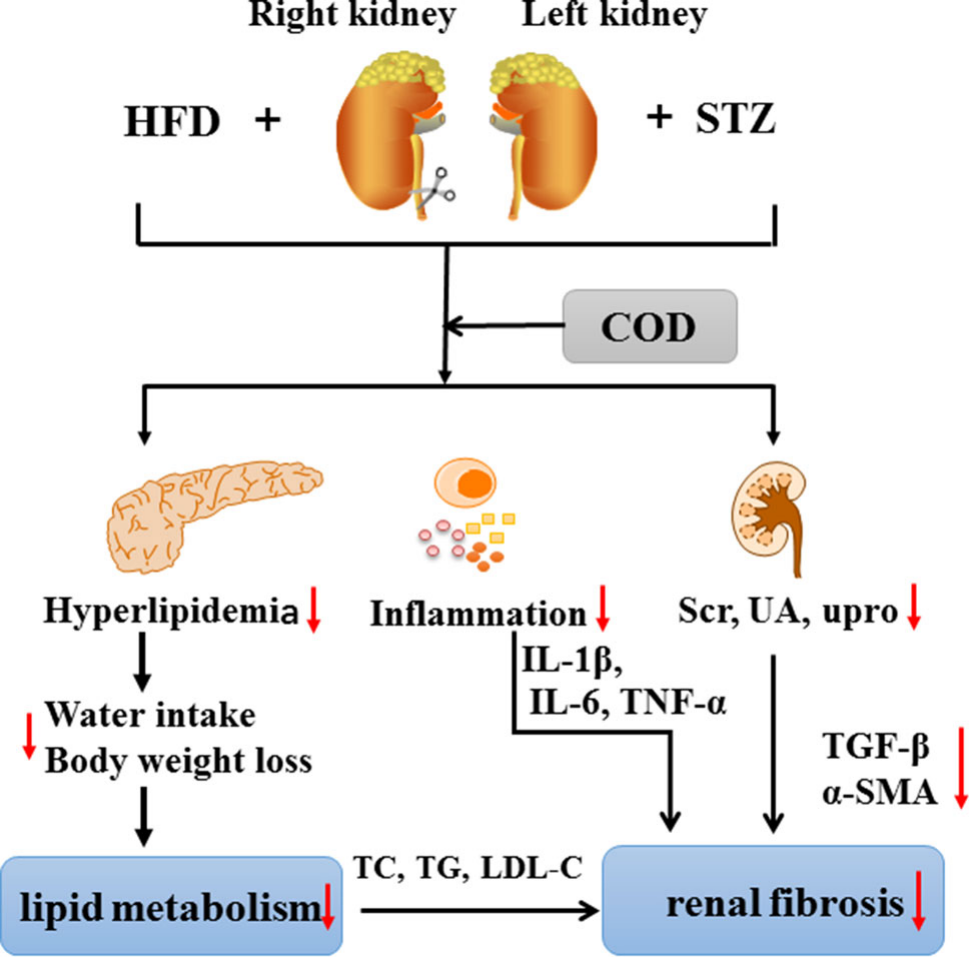

## Introduction

Diabetes has become the fourth cause of death because of non-communicable diseases (NCDs) in the world with the characteristic of abnormally high levels of glucose in the blood [1]. It is a lifelong disease and with its development, diabetic kidney disease (DKD) is the most notorious outcome, characterized with the glomerular basement membrane thickening, mesangial expansion, glomerular sclerosis, and/or renal tubular interstitial fibrosis, further leading to end-stage renal disease, which seriously affects people’s health and quality of life [2, 3]. Previous studies have demonstrated that the pathogenesis of DKD is predominantly mediated by non-enzymatic glycation, polyol pathway activation, protein kinase C activation, dyslipidemia, hypertension, glomerular hyperfiltration, oxidative stress, vasoactive substances, pro-inflammatory cytokines, and several other environmental factors [3, 4]. To date, the ultimate treatment of DKD is prevention and the following measures should be undertaken in all of the patients with diabetes with or without DKD according to clinical medicine guidelines, including stabilization of blood glucose concentration, strict blood pressure control, correction of lipid metabolism disorders, drug administration, and replacement of kidney therapy [5]. The traditional Chinese medicine theory revealed that the pathogenesis of DKD was closely related to the disorder of Yin and Yang in the body. Yin deficiency, blood stasis, and phlegmatic dampness were the pathogenesis of DKD [6].

*Codonopisis tangshen* Oliv. (COD) belongs to *Campanulaceae* family, which widely distributed in Central and East Asia [7]. It contains active components, such as polysaccharides, saponins and alkaloids, but few literatures on its pharmacological activities were reported in the past [8]. COD together with *C. pilosula* (Franch.) Nannf. and *C. pilosula* Nannf. var. *modesta* (Nannf.) L.T.shen, has been recorded as the source of *C. Radix* in Chinese pharmacopoeia (2015 edition), which plays a role in invigorating the spleen and nourishing [9]. Previously, we analyzed the prescription for the treatment of nephropathy using the Chinese traditional medicine database, and 80 in 164 prescriptions were contained *C. Radix*, indicating it played an important role in the treatment of nephropathy disease (data not published). However, *C. pilosula* (Franch.) Nannf was the most used in herbal formulae in China [10]. The evidence of therapeutical effect for COD was rare. Recent reviews have noted the compounds in COD and *C. pilosula* (Franch.) Nannf were obviously different [8]. Hence, it is very essential to provide positive proof for the application of COD in nephropathy disease.

Therefore, the melioration effect of COD was investigated on a DKD model induced by unilateral nephrectomy (UN)-high fat diet feeding (HFD) combined with streptozotocin (STZ), aiming to provide a reference for its clinical application.

## Results

### Effect of COD on Bodyweight, Water Intake, and Fasting Blood Glucose

**Fig. 1:**
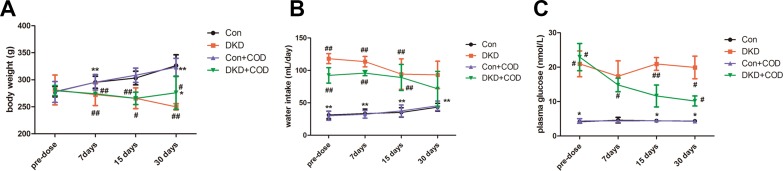
Effect of COD on body weight, water intake, and fasting blood glucose of DKD model (n = 6). All data were presented as the mean ± standard deviation. ^##^*p* < 0.01, ^#^*p* < 0.05 vs. Con group; **p < 0.01, *p < 0.05 vs. COD treatment

As shown in Fig. 1, DKD rats presented a significant body weight loss and water intake increase as compared to the control group. The average fasting blood glucose level of the DKD rats was higher than 17.37 mmol/L within 30 days. However, after 4 weeks of the COD treatment, the body weight loss obviously improved (275.92 ± 30.9 vs. 249.98 ± 5.78 g) and the water intake reduced (71.66 ± 26.87 vs. 93.00 ± 21.23 mg/day). In addition, the fasting blood glucose decreased to less than 11.1 mmol/L, indicating the hypoglycemic effect of the COD extract in the DKD rat model.

### Effect of COD on Renal Function

**Fig. 2:**
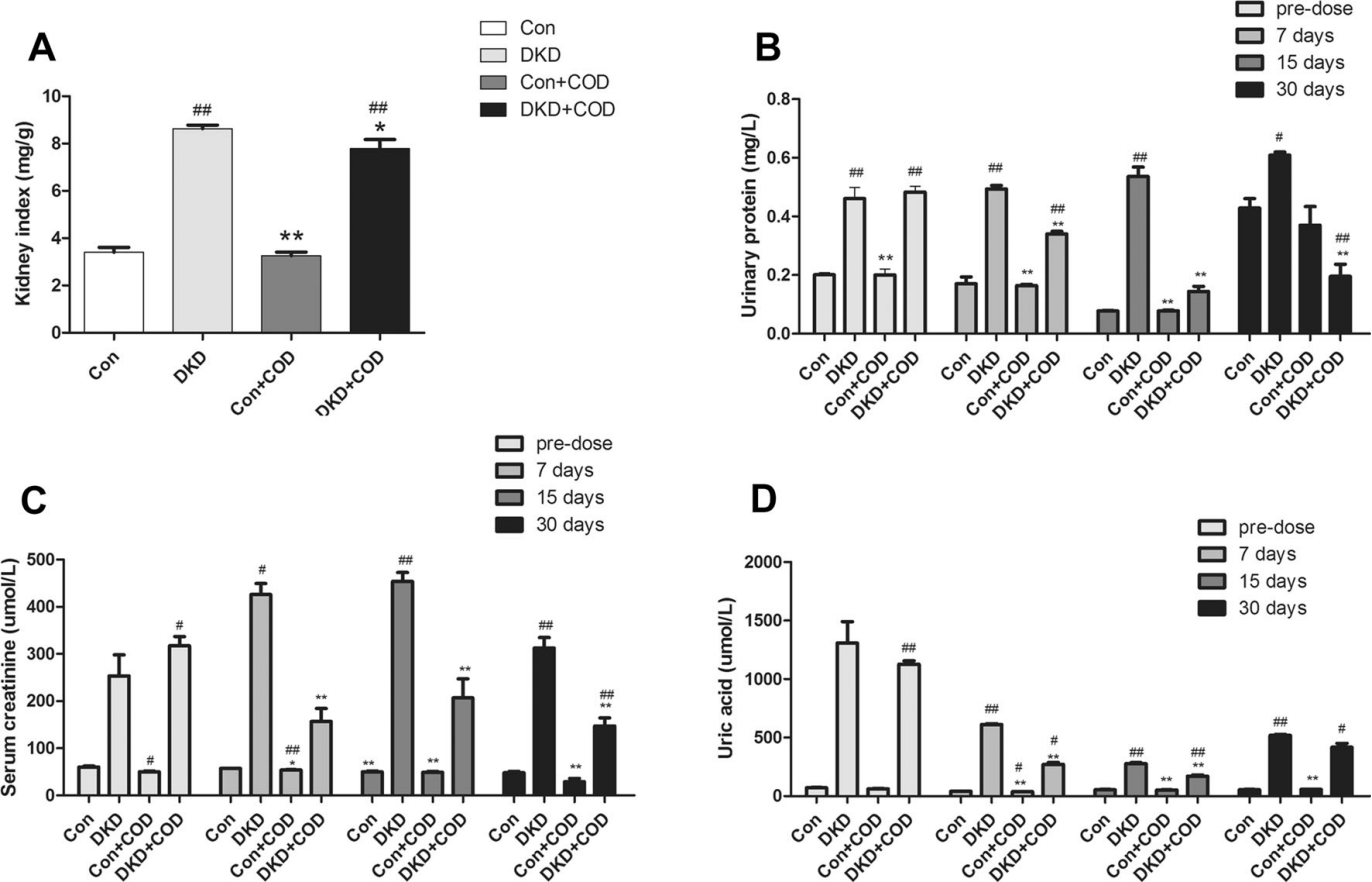
Effect of COD on hypertrophy of remnant kidney and renal function in rats. The kidney index was calculated by using the remnant kidney to body weight ratio (**a**). Urinary protein content (**b**) of 12 h. Serum levels of serum creatinine (**c**) and uric acid (**d**). All data were presented as the mean ± standard deviation (n = 6). ^##^*p* < 0.01, ^#^*p* < 0.05 vs. Con group; *p < 0.05, **p < 0.01 vs. COD treatment

During the experiment, the remnant kidney in the DKD rats was increased 2.53-fold than that of control group, but the hypertrophy of the kidney was inhibited after the COD treatment compared with DKD group. The levels of Scr, UA, and upro were tested three times after the COD treatment (Fig. 2), and they were increased to 3.19-fold, 17.25-fold and 1.29-fold in the DKD model compared with the control groups, respectively; in particular, a considerably large increase in the UA concentration was observed. Whereas the Scr, UA and upro levels were 0.53–0.63-fold, 0.19–0.53-fold and 0.3–0.73-fold decrease after COD treatment for 7, 15 and 30 days, respectively, suggesting that the renal function was improved after 4 weeks of COD treatment.

### Effect of COD on Renal Histology

**Fig. 3:**
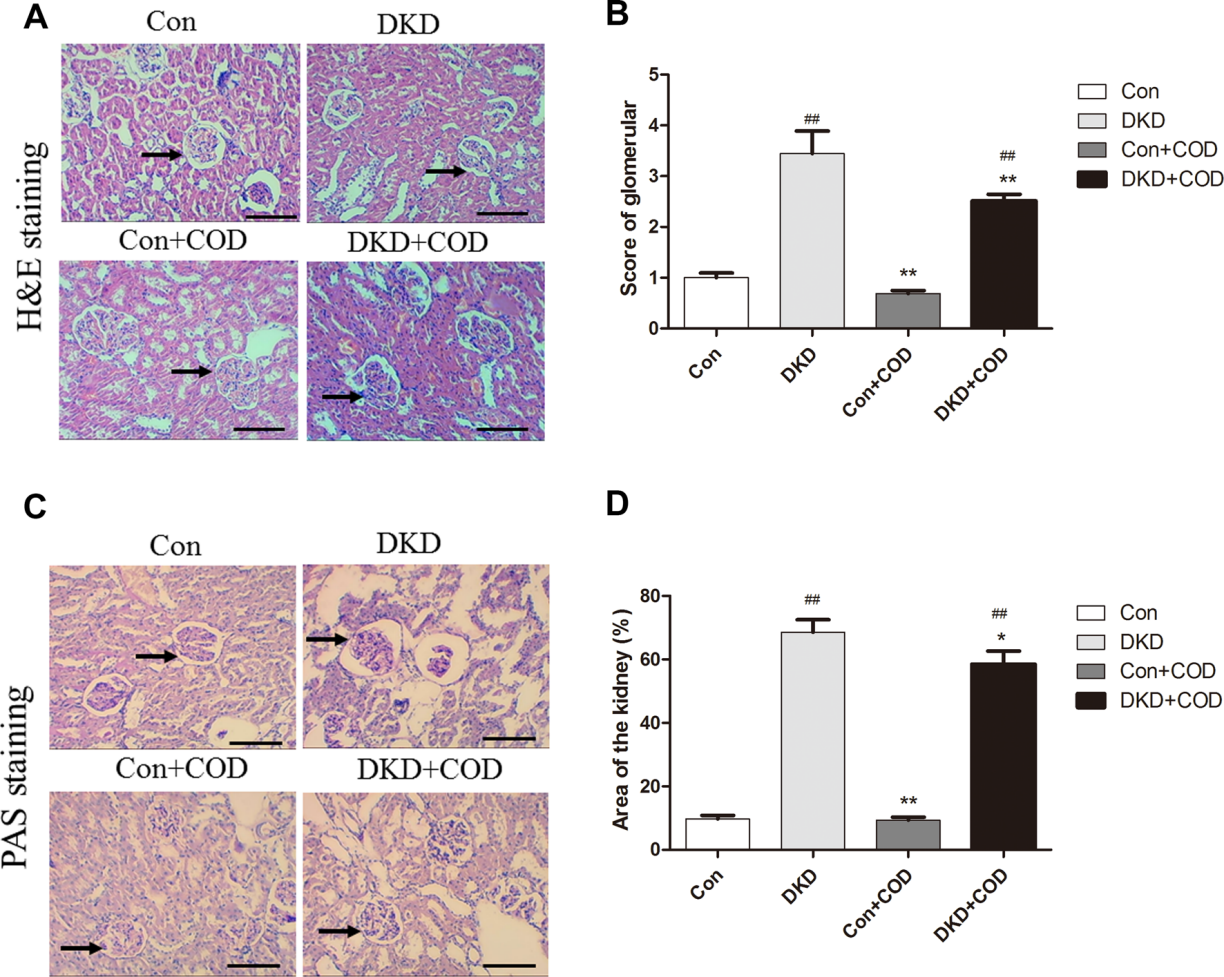
Effect of COD on renal histopathological alterations in DKD rats. The photomicrographs of H&E-stained (**a**) and PAS-stained (**c**) kidney sections were obtained for the four groups on day 30. Scale bar: 600 μm (×100). **b** Histopathological alterations in H&E-stained kidney slides were scored for the four groups on the basis of the semi-quantitative percentage of the damaged area on a scale from 0 to 4: 0, normal; 1, changes < 25% of cortical area; 2, changes 25–50% of cortical area; 3, changes 50–75% of cortical area; 4, changes > 75% of cortical area. **d** Score results of the glomerular area of the four groups. Magnification: ×200. Glomerular area was presented as the mean ± SD. ^##^*p* < 0.01, ^#^*p* < 0.05 vs. Con group; *p < 0.05, **p < 0.01 vs. COD treatment (n = 6)

In DKD groups, obvious pathological alterations of kidneys, such as remarkable glomerular hypertrophy, basement membrane thickening, mesangial expansion and glomerular sclerosis, were observed by H&E staining and PAS staining in the DKD kidneys (Fig. 3). However, this histological change was improved with the COD treatment and the score of glomerulus injury was significantly reduced as compared to that in the DKD group. The glomerular areas in the kidneys of the DKD group were significantly larger than those of the control groups, whereas the increase was attenuated after the COD treatment.

### Effect of COD on Pancreas Histology

**Fig. 4:**
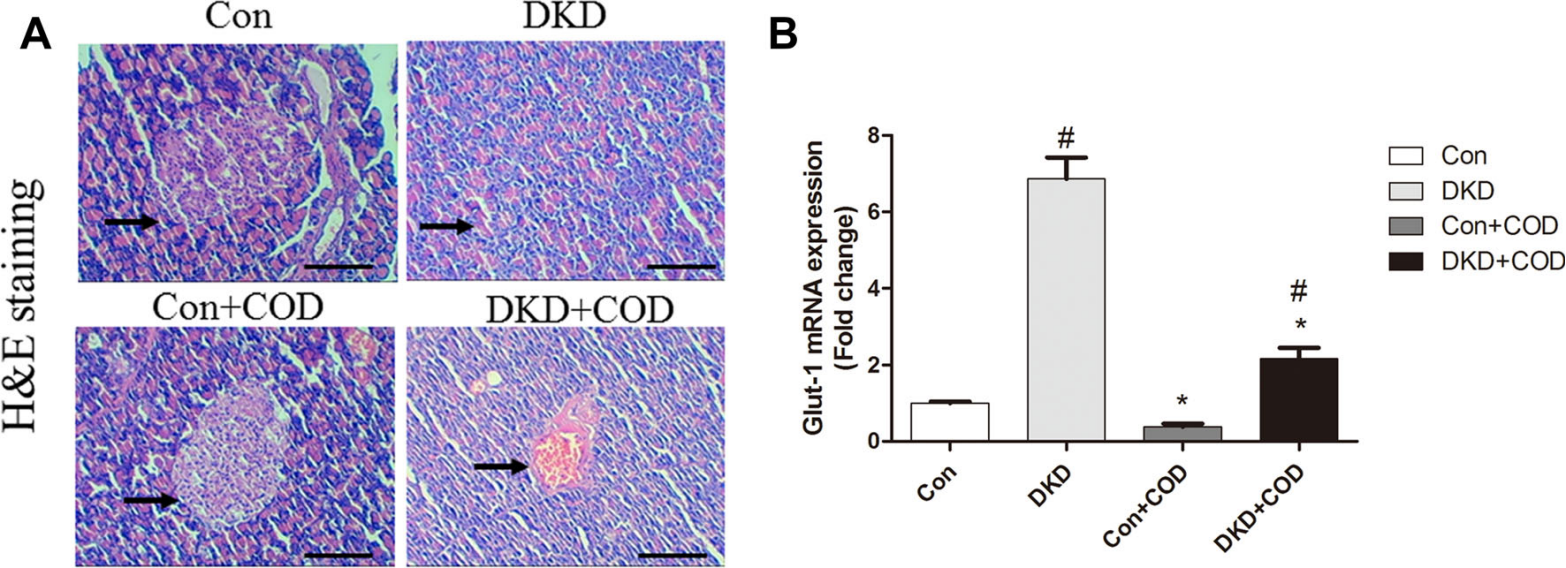
Effect of COD on pancreas and Glut-1 expression. The photomicrographs of H&E-stained sections (**a**) were obtained for the four groups on day 30 after COD treatment. Scale bar: 600 μm (×100). qPCR analysis of Glut-1 (**b**) expression in remnant kidney. All data were presented as the mean ± SD. ^##^*p* < 0.01, ^#^*p* < 0.05 vs. Con group; **p < 0.01 vs. COD treatment (n = 6)

The pathological alteration of the pancreas was examined on the basis of the H&E staining. Figure 4a showed significant reduction in the number of islet cells and increase in the infiltration of inflammatory cell in the DKD rats compared with the control groups. However, COD exhibited a protective effect on the islet cells, which secreted insulin to lower the blood glucose. In addition, GLUT1, glucose transporter 1, was measured using qPCR (Fig. 4b). In our experiment, the mRNA expression of GLUT1 was 5.85-fold up-regulation in the DKD rats compared with that of control group, while the COD treatment was down-regulated 3.17-fold the gene transcriptional level of GLUT1, suggesting that COD lowered the blood glucose level probably via inhibiting the GLUT1 expression in mesangial cells.

### Effect of COD on Lipid Metabolism

**Fig. 5:**
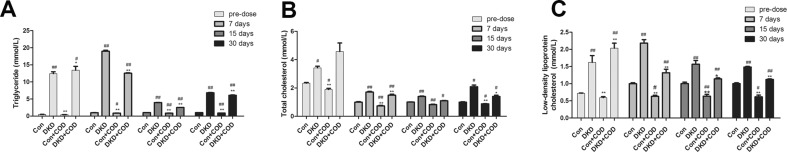
Effect of COD on lipid metabolism in DKD rats. Serum levels of triglyceride (**a**), total cholesterol (**b**), and low-density lipoprotein cholesterol (**c**) were measured on days 7, 15, and 30 after the COD treatment, respectively. All data were presented as the mean ± SD (n = 6). ^##^*p *< 0.01, ^#^*p* < 0.05 vs. Con group; *p < 0.05, **p < 0.01 vs. COD treatment

As shown in Fig. 5, the levels of TC, TG, and LDL-C were increased 1.46-fold, 25.3-fold and 2.24-fold in the DKD rats compared with that in control group, respectively. After 4 weeks of the COD extract treatment, the concentrations of TG, TC, and LDL-C were decreased to 0.66–0.89-fold, 0.67–0.87-fold and 0.6–0.76-fold but were still higher than in the control group, suggesting the COD extract reduced the lipid metabolism and the adipose deposition.

### Effect of COD on Inflammation

**Fig. 6:**
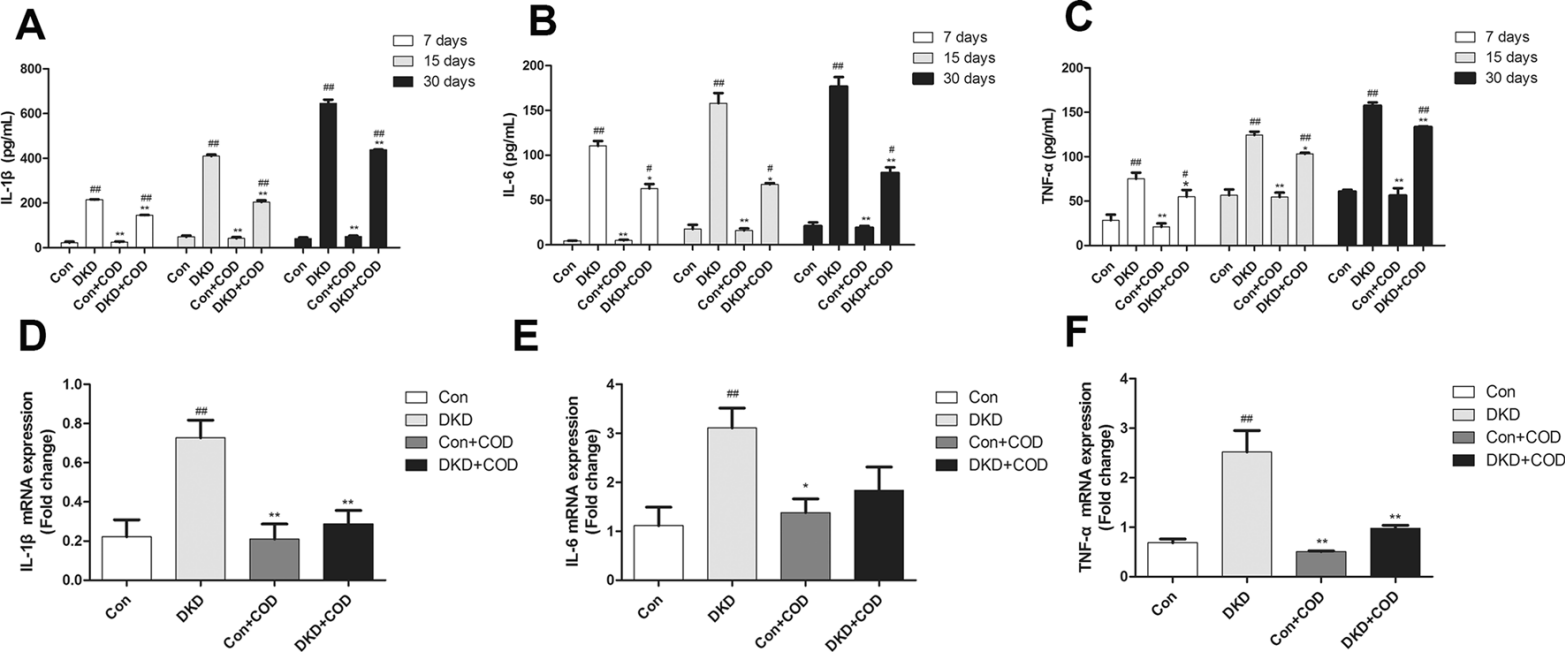
Effect of COD on inflammatory response in DKD rats. Serum levels of IL-1β (**a**), IL-6 (**b**), and TNF-α (**c**) were obtained by using ELISA on days 7, 15, and 30 after the COD treatment, respectively. The mRNA expressions of IL-1β (**d**), IL-6 (**e**), and TNF-α (**f**) in the remnant kidney were tested using qPCR on day 30 after the COD treatment. All data were presented as the mean ± SD (n = 6). ^##^*p* < 0.01, ^#^*p* < 0.05 vs. Con group; *p < 0.05, **p < 0.01 vs. COD treatment

To investigate whether the COD can relieve the inflammatory response, we examined the expression of the inflammatory mediator using ELISA and qPCR. As showed in Fig. 6, the contents of IL-1β, IL-6, and TNF-α were gradually increased in the DKD rats; whereas these increases were relieved by the COD treatment. In addition, we used quantitative qPCR to detect the mRNA expression of IL-1β, IL-6, and TNF-α. As results, the levels of IL-1β, IL-6, and TNF-α were markedly up-regulated in the DKD renal tissue, but they were decreased to 0.4–0.68-fold, 0.43–0.59-fold and 0.39–0.85-fold after the COD treatment for 7, 15 and 30 days, respectively. These findings suggested that inflammation was obviously induced and activated in the DKD rats, while COD ameliorated the inflammation by reducing the levels of the pro-inflammatory mediators.

### Effect of COD on Renal Fibrosis and Fibrosis Markers

**Fig. 7:**
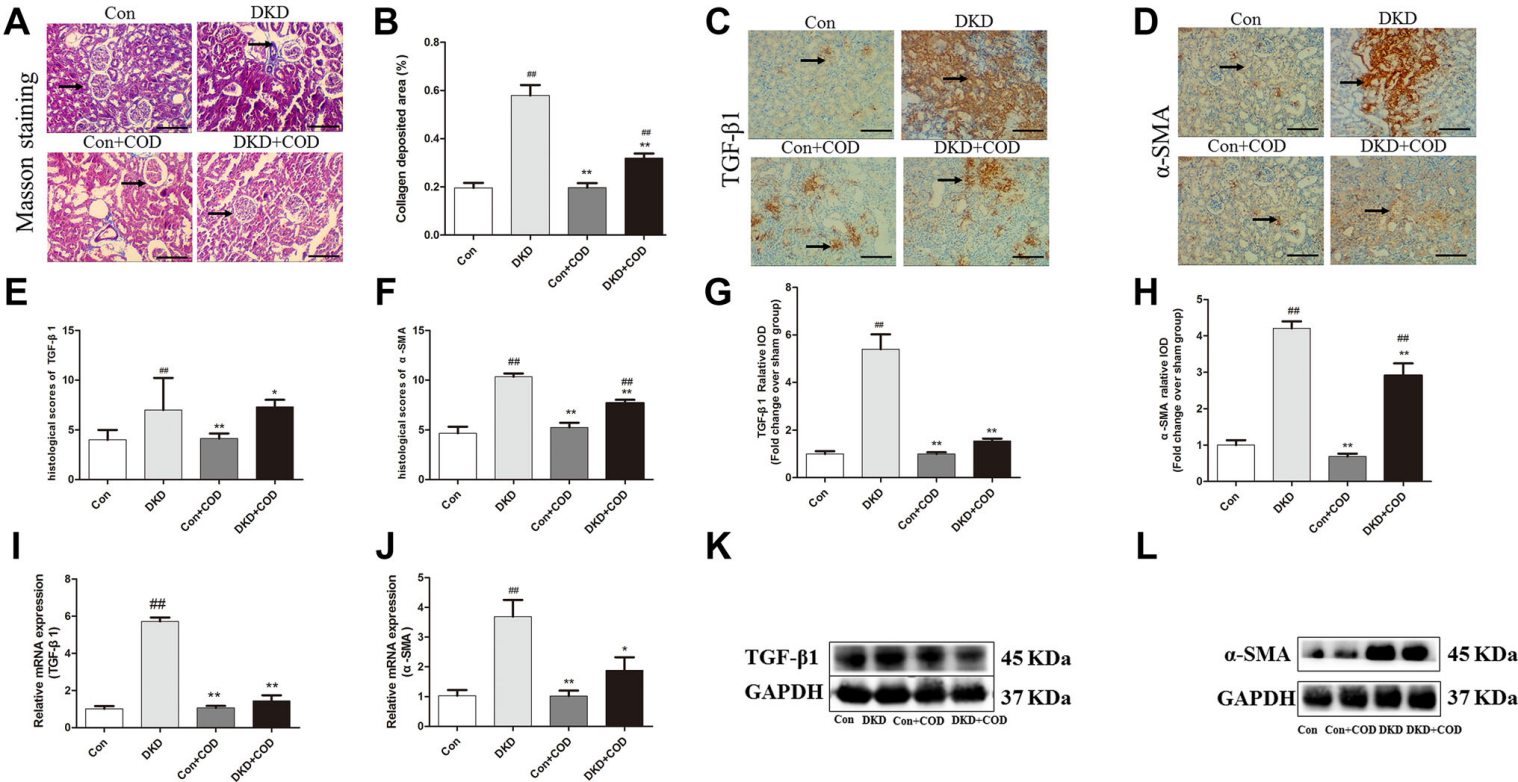
Effect of COD on inhibition of renal fibrosis and fibrosis markers in DKD rats. **a** The photomicrographs of Masson’s trichrome-stained kidney sections from the four experimental groups on day 30 after COD treatment. Scale bar: 600 μm (×200). **b** The collagen deposition area was assessed by Masson’s trichrome staining and was quantified using Image-Pro Plus 6.0 analysis software. The image and values are represented as mean ± SD. The expressions of TGF-β1 (**c**) and α-SMA (**d**) were determined by IHC staining in the kidney sections of rats from the four groups on day 30 after COD treatment. The histological score of TGF-β1 (**e**) and α-SMA (**f**) was evaluated in five horizons on a scale from 1 to 12: 0–3, negative (−); 4–6, low positive (+); 7–9, medium positive (++); and 9–12, strongly positive (+++). The quantitative determination of the renal TGF-β1 (**g**) and the α-SMA (**h**) protein content was performed using the Image-Pro Plus 6.0 software. The mRNA expressions of TGF-β1 (**i**) and α-SMA (**j**) were analyzed using qPCR in the remnant kidney. The proteins of TGF-β1 (**k**) and α-SMA (**l**) were examined by western blotting. All data were presented as the mean ± SD. ^##^*p* < 0.01; ^#^*p* < 0.05 vs. Con group; *p < 0.05; **p < 0.01 vs. COD treatment (n = 6)

The glomerular changes accompanied by a noticeable increase in collagen deposition were shown by a collagen-specific Masson’s trichrome staining. However, treatment with COD reduced collagen deposition by 45%. To investigate the anti-fibrotic effect of COD, immunohistochemistry was used to determine the protein expressions of TGF-β1 and α-SMA in the DKD kidneys (Fig. 7). Compared with the control groups, the DKD groups showed a significant overexpression of TGF-β1 and α-SMA, but the increases in the TGF-β1 and α-SMA expressions attenuated to a certain extent after the COD treatment. Meanwhile, the mRNA expression of TGF-β1 and α-SMA were tested to evaluate whether COD regulated their transcriptional levels. As results, the expression of TGF-β1 and α-SMA was increased by 4.6-fold and 2.6-fold in the DKD rats compared with the control groups, respectively. In contrast, the mRNA expressions of TGF-β1 and α-SMA were 0.75-fold and 0.49-fold down-regulation after the COD treatment. Western blot showed that the trend of protein levels of TGF-β1 and α-SMA were consistent with the mRNA expression.

## Discussion

Previously, the combination of UN–HFD–STZ has been proven to be a rapid and effective method to stimulate DKD in rats [11]. Progressive body weight loss and significative water intakes with blood glucose levels above 17.37 mmol/L were resulted from diabetes. The levels of Scr, UA, and upro were significantly increased, subsequently, glomerular basement membrane thickening, mesangial expansion, glomerular sclerosis, interstitial inflammation and collagen fibers increase were observed by H&E, PAS and MT staining, indicating that the DKD model was successfully established. However, the COD treatment significantly attenuated the diabetes symptoms and improved the renal function of the DKD rats. The histopathological findings were also ameliorated after the COD administration.

It is well accepted that hyperglycemia is the basic pathophysiological change in the tissue damage caused by DKD [12, 13]. Persistent hyperglycemia results in insulin deficiency or resistance, thus impairing glucose metabolism. There are two major types of glucose transporters, namely sodium dependent glucose transporter (SGLT) and glucose transporter (GLUT), to the regulation of the glucose metabolism in the kidney [14, 15]. GLUT1 is an insulin-insensitive glucose transporter. It was increased by 5.8-fold in mesangial cells in DKD rats compared with that in the control rats, while the COD treatment down-regulated the mRNA expression of GLUT1. A negative feedback mechanism probably played an important role in the regulation of glucose metabolism in the mesangial cells, but more research is needed.

Hyperlipidemia is another main cause of DKD. An excess of TC deposition result in the damage of renal mesangial cell [16, 17]. Previous studies have proved that endothelial dysfunction and the apoptosis of proximal tubule epithelial cells were associated with hyperlipidemia, which accelerate the progression of renal fibrosis and glomerular sclerosis [18, 19]. Clinical trials demonstrated that the down-regulation of the levels of TC, TG, and LDL-C effectively reduced the excretion of podocytes in UA and upro, thus improving the renal function. In our study, the levels of TC, TG, and LDL-C were higher in the DKD rats, while they were decreased after the 4 weeks COD treatment, indicating that COD improved the lipid accumulation and thereby ameliorated the DKD.

The inflammation activated many inflammatory cytokines (especially IL-1β, IL-6 and TNF-α) in DKD, and contributing to the progression of DKD [20, 21]. IL-1β is responsible for the excessive expression of chemotactic agents and adhesion molecules, leading to the inflammatory reaction in renal endothelium. IL-6 increases the membrane permeability and alters the extracellular matrix of the renal endothelium [2]. TNF-α activates of the transcription factors and growth factors of other inflammatory mediators, resulting in the changes of membrane permeability of the renal endothelium [2]. In our experiment, the expressions of IL-1β, IL-6, and TNF-α were up-regulated in the DKD group compared with those in the control rats within 30 days in a time-dependent manner, but these levels were decreased to some extent by the COD administration, indicating COD alleviation effect on the inflammatory response associated with the inhibition of the activation of the inflammatory cytokines (IL-1β, IL-6, and TNF-α).

Increasing evidence has shown that hyperglycemia promotes the expression of TGF-β1 in mesangial cells of the kidney and TGF-β1 promotes the expression of α-SMA, which are the biomarkers of fibrosis [22, 23]. The accumulation of fibrotic factors of the renal is a critical factor in the mechanism responsible for the progression of renal fibrosis [24–27]. In our study, the glomerular fibrotic lesions and collagen accumulation were observed in DKD kidneys. Meanwhile, the protein and mRNA expressions of TGF-β1 and α-SMA were significantly higher in the DKD rats than that in the control group. Conversely, COD extracts obviously down-regulated the TGF-β1 and α-SMA versus the model rats, indicating that COD alleviated renal fibrosis in DKD probably through inhibiting TGF-β1 signaling pathway.

In conclusion, the DKD model induced by UN–HFD–STZ was successfully established in this study. The COD extracts significantly improved the blood glucose metabolism and the lipid accumulation, inhibited the inflammatory response, and relieved renal fibrosis. However, because of the multiple compounds in COD, further research is needed to clarify the mechanism of the amelioration of COD on DKD and its main active compounds.

## Experimental Section

### Materials and Reagent

Streptozotocin (STZ) was purchased from Merck Life Science (Shanghai) Co., Ltd. (Shanghai, China). The PBS solution was obtained from Guangzhou Anfei Biotechnology Co. Ltd. (Guangzhou, China). The kits of serum total cholesterol (TC), triglyceride (TG), low-density lipoprotein cholesterol (LDL-C), serum creatinine (Scr), uric acid (UA), and urinary protein (upro) were purchased from Nanjing Jiancheng Bioengineering Institute (Nanjing, China). The ELISA kits for interleukin-6 (IL-6), IL-1β, and tumor necrosis factor-α (TNF-α) were purchased from Neobioscience Technology Company (Shenzhen, China). *Codonopsis tangshen* Oliv. (COD) was collected from the Shennongjia forestry region of Hubei Province and authenticated by Dr. Jingyu He (Guangzhou Institute of Advanced Technology, Chinese Academy of Sciences, Guangzhou, China).

### Animals

Six-week-old male Sprague–Dawley rats (190–210 g, certification: SCXK-Yue-2016-0041) were provided by the Laboratory Animal Center of Southern Medical University (Guangzhou, China). In the whole experiment, all of the animals were treated according to the National Institutes of Health guidelines for the Care and Use of Laboratory Animals (8th Edition, 2011). All of the animals were housed in an air-conditioned room at 23 °C ± 2 °C and 40% ± 5% relatively humidity under an alternating day and night cycle per 12 h and kept with free food and water.

### COD Preparation

Dried and ground COD (50 g) was extracted with 500 ml of 75% ethanol (*v/v*) three times, 1 h each time. After collecting all of the extraction solutions, the sample was evaporated under vacuum (Tokyo Rikakikai Co., Ltd., Tokyo, Japan) at 60 °C to yield the concentrate with a relative density of 1.15 g/mL. After adding 250 mL of purified water and 0.2 g of active carbon powder to the concentrate, we boiled the mixture for 10 min. The supernatant was obtained by centrifugation at 7000 g. Then, the supernatant was added to the drinking water with a concentration of 0.5 g/mL for oral administration to the animals. For quality control this preparation, a previous method was used to determine the content of tangshenoside I as follows: a YMC-Pack Pro-C18 column (4.6 mm × 250 mm, 5 μm, YMC Co., Ltd., Japan) with column temperature at 30 °C; a binary eluent of acetonitrile (A) and 0.1% (v/v) phosphoric acid (B) as mobile phase with gradient conditions: 0–10 min, 98–92% B; 10–35 min, 92–80% B; 35–50 min, 80–70% B; 50–60 min, 70–50% B; 60–65 min, 50–10% B.The flow rate was 1.0 mL/min and detection wavelength was 215 nm [28, 29]. The content of tangshenoside I was 2.67 ± 0.06 mg/mL.

### Animal Experiment

Twenty-four male SD rats were randomly divided into four groups (n = 6 for each group): Control (Con), DKD model, Con + COD, and DKD + COD groups. In the experiment, the SD rats were fed either a basal diet (34% corn flour, 32% wheat flour, 13% wheat bran, 17% soybean meal, 4% fish meal, and 1% salt) or a high-fat diet (HFD, 66.5% basal diet, 10% lard, 20% sucrose, 1.5% cholesterol, 1% bile acid, and 1% yolk powder) for 11 weeks. COD administration at a dose of 2.7 g/kg was calculated from a commonly used clinical dose of 30 g/day for the treatment of diabetes [30, 31]. The operation was performed according to previous studies [32, 33]. After the rats were anesthetized with ketamine injected intramuscularly at a dose of 100 mg/kg [34], nephrectomy of the right kidney was performed after ligating the renal hulum with 5-0 sterile sutures. When the rats woke up, they were administered an intraperitoneal injection of penicillin (5000 units/each rat) for two consecutive days to prevent infection. On the second postoperative day, all of the rats (except those in the control groups) were given a high-fat diet continuously. Four weeks later, the rats were intraperitoneally injected with STZ at a dose of 40 mg/kg in citrate buffer, pH 4.5. After 2 h of administration, the high-fat diet feeding was continued. The fasting blood glucose or the random blood glucose was monitored on days 3, 7, 14, and 21 after the STZ injection. As the levels of blood glucose were higher than 16.8 mmol/L, the model was considered a diabetic model. Further, the renal function indices (Scr, UA and upro), were higher than those in the control group, indicating that the DKD model was successfully established. Rats with were excluded from the experiment. The DKD rats were randomly divided into two groups: DKD group and DKD + COD group. The COD extract was oral administered to the COD treatment group at the dose of 2.7 g/kg/day for 4 weeks, while the purified water was to the control group at the same dose.

### Sample Preparation

The serum of each group was obtained from the orbit on days 0, 7, 15, and 30 after the STZ administration for 1 month to measure the concentrations of UA, Scr, TG, TC, LDL-C, IL-1β, IL-6, and TNF-α according to the manufacturer’s instructions. Further, the urine was collected in a metabolic cage for a period of 12 h to detect the content of upro. All of the samples were stored at −20 °C. All of the rats were sacrificed with ketamine injected intramuscularly at a dose of 100 mg/kg after 11 weeks [34]. The kidneys from each group were weighed to calculate the kidney index (kidney weight/body weight, mg/g). Then, the kidney was cut into two parts: one was fixed in 10% formalin for the hematoxylin and eosin (H&E), periodic acid–Schiff (PAS), Masson’s trichrome staining (MT) and immunohistochemical (IHC) staining; the other was stored in liquid nitrogen for the detection of RNA and protein. The pancreas was fixed in 10% formalin for H&E staining.

### H&E Staining

The renal and pancreatic tissues were embedded in paraffin. Four-mm-thick sections were deparaffinized and stained according to the standard procedures [35]. The extent of glomerular sclerosis was graded on a scale from 0 to 4: 0, normal; 1, changes < 25% of cortical area; 2, changes 25–50% of cortical area; 3, changes 50–75% of cortical area; 4, changes > 75% of cortical area [30]. The mean scores were calculated and determined on five randomly chosen fields at a magnification of 200×. The decrease of islet cell and the infiltration of inflammatory cell were regarded as the indicators to assess the pancreas.

### Masson’s Trichrome Staining

Paraffin-embedded kidney tissues were sliced, dewaxed, and stained by Masson’s Trichrome. The degree of renal fibrosis was assessed based on the amount of collagen deposition (blue color area over the whole cortex area) using an optical microscope (Olympus) and quantification of collagen was analyzed using Image-Pro Plus 6.0 software (Media Cybernetics, Bethesda, MD, USA). For each group, five fields were analyzed at magnification of 200×. The areas of fibrotic lesion were expressed as a percentage of fibrotic area relative to the entire area [35].

### PAS Staining

Renal tissues were embedded in paraffin and sectioned at 4 mm. Sections were stained with PAS according to previous study to evaluate the pathological changes in the renal structure in the DKD rats [26]. The glomerular area was analyzed using the Image-Pro Plus 6.0 software (Media Cybernetics, Bethesda, MD, USA)

### IHC Staining

Paraffin-embedded kidney tissues were baked at 60 °C for 3 h and deparaffinized in xylene for 15 min three times. Next, the sections were blocked with 3% (*v/v*) H_2_O_2_ for 20 min and washed thrice with the PBS solution for 5 min. Non-enzymatic antigen retrieval was performed by heating the sections to 92 °C-95 °C in the citrate buffer (10 mM, pH 6.0) for 15 min and washed three times with PBS for 5 min. Then, the sections were incubated with the primary antibody overnight at 4 °C, rewarmed to room temperature, and washed three times with PBS for 5 min [36]. The primary antibodies were used as follows: rabbit monoclonal antibody against α-smooth muscle actin (α-SMA) (1:100 dilution, BIOSS, Beijing) and transforming growth factor-β1 (TGF-β1) (1:100 dilution, Boster Biological Technology Co. Ltd, USA). The secondary antibody incubation was performed at 37 °C for 20 min and washed three times with PBS. Immunostaining was performed with 3,3-diaminobenzidine (DAB). The sections were counterstained with hematoxylin and finally sealed using neutral gum. Photomicrographs of 200-fold-magnified sections (n = 6 per animal) were taken using an optical microscope (Olympus BX41, 400×) and analyzed with the Image-Pro Plus 6.0 analysis software (Media Cybernetics, Bethesda, MD, USA). The brown area was considered positive, and the staining intensity was calculated as the integral optical density (IOD). For the semi-quantitative analysis, 5 randomly collected IODs of the same area for each section were measured. The calculation of the mean percentage of positive cells was scored as follows: the percentage of positive cells in 0% was counted 0; the percentage of positive cells in 1–25% was counted 1; 26–50% was counted 2; 51–75% was counted 3; ≥ 76% was counted 4. The staining intensity was scored as 0 (negative), 1 (weak), 2 (moderate) and 3 (strong). The final histological scores was assessed by multiplying the score for the percentage of positive cells and the staining intensity and graded on a scale from 1 to 12: negative (−) for scores of 0–3, low positive (+) for scores 4–6, medium positive (++) for scores 7–9 and strongly positive (+++) for scores 9–12 [36].

### RNA Reverse Transcription and Quantitative Real-Time PCR (qPCR)

The total RNA was extracted from each renal tissue using RNAprep Pure Tissue Kit (Tiangen Biotech, Guangzhou, China) according to the manufacturer’s protocol and reverse transcribed into the first-strand cDNA which was synthesized from 500 ng total RNA and 5× primescript RT Master Mix 2 μL (Takara Bio INC., Kusatsu, Japan) in a 10 μL reaction volume, and then, amplified by the PCR reactions using GoTaqR qPCR Master Mix, 2X (Promega Corporation, Madison, USA), primers of TGF-β1, α-SMA, Glut-1, GAPDH, IL-1β, TNF-α and IL-6 (designed and synthesized by Suzhou Hongxun Biological Co., Ltd.; Table 1, [37–40]), and RNase-free ddH_2_O in a 10 μL reaction volume using Applied Biosystems^®^ 7500 Fast Real-time PCR System (Thermo Fisher Scientific, USA) under the following reaction conditions: 50.0 °C for 3 min and 95.0 °C for 3 min, followed by 40 cycles at 95.0 °C for 10 s and 60.0 °C for 30 s. The threshold cycle (Ct) was recorded by the instrument’s software (7500 Fast System Software version v2.3), and the fold changes in the mRNA expression were calculated according to the comparative Ct method (2^−ΔΔCT^) as presented.

**Table 1:** Primer sequences for quantitative real-time PCR amplification

Name	Primer forward	Primer reverse	Reference
TGF-β1	CGTCAGACATTCGGGAAGC	CAGCCACTCAGGCGTATCA	[37]
α-SMA	ACTGGGACGACATGGAAAAG	GTTCAGTGGTGCCTCTGTCA	[38]
Glut-1	GGTGTGCAGCAGCCTGTGTA	GACGAACAGCGACACCACAGT	[39]
GAPDH	GATGGTGAAGGTCGGTGTG	ATGAAGGGGTCGTTGATGG	[38]
IL-1β	CACCTCTCAAGCAGAGCACAG	GGGTTCCATGGTGAAGTCAAC	[23]
IL-6	CCAAGACCATCCAACTCATCTTG	CACAGTGAGGAATGTCCACAAAC	[23]
TNF-α	CCAGGTTCTCTTCAAGGGACAA	CTCCTGGTATGAAATGGCAAATC	[40]

### Western Blot

The remant kidney tissues of all rats were lysed with RIPA buffer (APEXBIO, USA) in the presence of cocktail protease inhibitor (APEXBIO, USA) in an ice bath, and were homogenized with a homogenizer. After the homogenate was centrifuged at 12000 rpm for 30 min, the supernatant was used to measure protein concentration by using a bicinchoninic acid (BCA) assay kit (Beyotime Biotechnology, Shanghai, China), and then adding 5× loading buffer to supernatant for western blotting. All protein samples were loaded onto 10% sodium dodecyl sulfate–polyacrylamide gel electrophoresis (SDS-PAGE) for 1.5 h and then transferred to poly-vinylidene fluoride membrane. The membrane was blocked with 5% nonfat milk in Tris-buffered saline with Tween-20 (TBS-T) for 2 h at room temperature, and then incubated overnight with the primary antibodies, including TGF-β1 (1:800; affinity), α-SMA (1:800; affinity) and GAPDH (1:3000; affinity), at 4 °C. After washing three times, the membranes were incubated with the secondary antibody (1:2000) for 1 h at room temperature and then were washed three more times. Finally, the blots were detected using the enhanced chemiluminescence (ECL) method, and target band molecular weight and net optical density were analyzed using a gel image processing system (FluorChem R, ProteinSimple, USA).

### Statistical Analysis

All of the data were analyzed using a Graphpad prism (Graphpad Software, San Diego, CA) and presented as mean ± SD. The statistical differences (*p *< 0.05 and *p* < 0.01) among the groups were obtained using one-way ANOVA followed by Tukey’s multiple comparison test with SPSS version 20.0 statistical software (IBM INC., New York, USA).

## References

[1] World Health Organization (2017). In the WHO Document Production Services.

[2] Behl T, Kaur I, Goel H, Pandey RK (2014). World J. Pharm. Pharm. Sci..

[3] Ge J, Miao JJ, Sun XY, Yu JY (2016). J. Ethnopharmacol..

[4] Han PX, Shao MM, Guo L, Wang WJ, Song GF, Yu XW, Zhang CL, Ge N, Yi TG, Li SM, Du H, Sun HL (2018). Am. J. Transl. Res..

[5] P.B. Mark, P. Winocour, ABCD-RA Clinical Practice Guidelines—Lipid Management in DN &/or DM CKD (The Renal Association, Bristol, 2017), pp. 1–26

[6] Du YG, Zhang KN, Gao ZL, Dai FJ, Wu XX, Chai KF (2018). Exp. Ther. Med..

[7] Bi HY, Zhang LP, Chen Z, Wu B (2008). J. Chin. Mater. Med..

[8] He JY, Ma N, Zhu S, Komatsu K, Li ZY, Fu WM (2015). J. Nat. Med..

[9] International Pharmacopoeia Commission (2015). Pharmacopoeia of People’s Republic of China.

[10] Shergis JL, Liu S, Chen X, Zhang AL, Guo X, Lu C, Xue CC (2015). Phytother. Res..

[11] Park KM, Hussein KH, Nam HS, Kim HM, Kang BM, Lee DG, Han HJ, Woo HM (2016). Lab Anim..

[12] Hong N, Lee M, Park S, Lee YH, Jin SM, Kim JH, Lee BW (2018). Sci. Rep..

[13] Almalki AL, Sayed AA, El Rabey HR (2013). Evid. Based Complement Alternat. Med..

[14] Girard J (2017). Nephrol. Ther..

[15] Wasik AA, Lehtonen S (2018). Front. Endocrinol. (Lausanne).

[16] Y. Katsuda, Y. Kemmochi, M. Maki, R. Sano, Y. Toriniwa, Y. Ishii, K. Miyajima, K. Kakimoto, T. Ohta, J. Diabetes Res. 1–6 (2014) 10.1155/2014/363126 PMC4142530 25177706

[17] Katsuda Y, Kemmochi Y, Maki M, Sano R, Toriniwa Y, Ishii Y, Miyajima K, Kakimoto K, Ohta T (2014). J. Diabetes Res..

[18] Yakush WJK (2017). Nurs. Clin. N. Am..

[19] Fan Y, Zhang J, Xiao W, Lee K, Li Z, Wen J, He L, Gui D, Xue R, Jian G, Sheng X, He JC, Wang N (2017). Sci. Rep..

[20] Wu W, Yang JJ, Yang HM, Huang MM, Fang QJ, Shi G, Mao ZM, Han WB, Shen SM, Wan YG (2017). Int. J. Mol. Med..

[21] Alicic RZ, Johnson EJ, Tuttle KR (2018). Adv. Chronic Kidney Dis..

[22] Vallée A, Lecarpentier Y, Guillevin R, Vallée JN (2017). Oncotarget..

[23] Peng H, Wang Q, Lou T, Qin J, Jung S, Shetty V, Li F, Wang Y, Feng XH, Mitch WE, Graham BH, Hu Z (2017). Nat. Commun..

[24] Wu Su-Zhen, Yang Si-Jun, Chen Hong-Min, Peng Fang-Fang, Yu Hong, Krepinsky Joan C., Zhang Bai-Fang (2017). Dual roles of parathyroid hormone related protein in TGF-β1 signaling and fibronectin up-regulation in mesangial cells. Bioscience Reports.

[25] Liu S, Ye L, Tao J, Ge C, Huang L, Yu J (2018). Pharm. Biol..

[26] Kim TW, Kim YJ, Seo CS, Kim HT, Park SR, Lee MY, Jun JY (2016). Phytomedicine..

[27] Dua CY, Ren YZ, Yao F, Duan JL, Zhao HE, Du YX, Xiao X, Duan HJ, Shi YH (2017). Int. J. Biochem. Cell Biol..

[28] J.Y. He (Division of Pharmacognosy, Institute of Natural Medicine University of Toyama, 2013), pp. 1–96

[29] He JY, Zhu S, Komatsu K (2014). Phytochem. Anal..

[30] X.L. Tong, W.K. Liu, Y. Zhai, Z. Zhen, B. Chang, H.Y. Ji, Cross-strait conference on the Development of Traditional Chinese Medicine (2009)

[31] Y.P. Zhang (In People’s Medical Publishing House Co., Ltd., Beijing, 1996), p. 238

[32] Uil M, Cantlebery AMLS, Butter LM, Larsen PWB, de Boer OJ, Leemans JC, Florquin S, Roelofs JJTH (2018). Sci. Rep..

[33] Chang CC, Chen YC, Huang HC, Lee FY, Chang FY, Lin HC, Chan CY, Wang SS, Lee SD (2006). J. Chin. Med. Assoc..

[34] Li A, Zhang X, Shu M, Wu M, Wang J, Zhang J, Wang R, Li P, Wang Y (2017). Phytomedicine..

[35] Sun H, Ge N, Shao M, Cheng X, Li Y, Li S, Shen J (2013). Diabetes Res. Clin. Pract..

[36] Zeng CY, Chen TT, Zhang Y, Chen Q (2017). J. Cancer..

[37] Lo CS, Shi Y, Chang SY, Abdo S, Chenier I, Filep JG, Ingelfinger JR, Zhang SL, Chan JS (2015). Diabetologia..

[38] Anders HJ, Suarez-Alvarez B, Grigorescu M, Foresto-Neto O, Steiger S, Desai J, Marschner JA, Honarpisheh M, Shi C, Jordan J, Müller L, Burzlaff N, Bäuerle T, Mulay SR (2017). Kidney Int..

[39] Marotta D, Karar J, Jenkins WT, Kumanova M, Jenkins KW, Tobias JW, Baldwin D, Hatzigeorgiou A, Alexiou P, Evans SM, Alarcon R, Maity A, Koch C, Koumenis C (2011). Cancer Res..

[40] Xue XT, Kou XX, Li CS, Bi RY, Meng Z, Wang XD, Zhou YH, Gan YH (2017). Sci. Rep..

